# Physical Structural Mechanical and Thermal Insulation Properties of Hemp Fiber-Substituted Geopolymer Composites

**DOI:** 10.3390/ma18112536

**Published:** 2025-05-28

**Authors:** Ahmet Filazi, Reyhan Akat, Muharrem Pul, Songül Tortuk, Ali Özdin

**Affiliations:** 1 Department of Construction, Kırıkkale Vocational School, Kırıkkale University, Kırıkkale 71450, Türkiye; ahmetfilazi@kku.edu.tr; 2 Department of Architecture, Faculty of Engineering and Architecture, Yozgat Bozok University, Yozgat 66900, Türkiye; 3 Department of Electrical and Energy, Kırıkkale Vocational School, Kırıkkale University, Kırıkkale 71450, Türkiye; mpul@kku.edu.tr; 4 Department of Engineering Sciences, Middle East Technical University, Ankara 06800, Türkiye; trtk_sngl@hotmail.com; 5 Institute of Science, Kırıkkale University, Kırıkkale 71450, Türkiye; ozdinnali@gmail.com

**Keywords:** geopolymer, hemp fiber, blast furnace slag, mechanical properties, thermal insulation, high-temperature resistance

## Abstract

This study examines the thermal insulation capacity, mechanical performance, and high-temperature resistance of geopolymer composites reinforced with 5%, 10%, and 20% hemp fiber. This research aims to develop sustainable, high-performance construction materials with enhanced thermal efficiency and structural integrity. Thermal conductivity, compressive strength, and flexural strength tests were conducted on geopolymer mortar specimens to evaluate their performance. The results indicate that increasing hemp fiber content improves thermal insulation, with the 20% hemp fiber mixture achieving the lowest thermal conductivity. However, hemp fiber reinforcement leads to reductions in both compressive and flexural strength while maintaining structural stability. These findings highlight the potential of hemp fiber-reinforced geopolymers as eco-friendly alternatives to conventional insulation materials, particularly for applications requiring fire resistance and thermal efficiency, despite the observed decrease in mechanical properties. This research contributes to the advancement of sustainable construction materials and underscores the viability of hemp fiber-reinforced geopolymer mortars for industrial applications. Further studies are recommended to optimize mix designs and assess long-term durability under various environmental conditions.

## 1. Introduction

Environmental sustainability is gaining increasing importance worldwide, and renewable resources play a critical role in addressing contemporary environmental challenges and promoting long-term sustainability [1,2]. Cement production alone is responsible for approximately 7–8% of global CO_2_ emissions [3], highlighting the need for the development of more environmentally friendly and sustainable construction materials. In this regard, several studies have explored various eco-friendly substitutes, such as waste materials like fly ash, bottom ash, steel slag, and glass, which can be incorporated into concrete to reduce environmental impact [4]. Furthermore, using quarry waste as an alternative filler in ultra-high-performance cement pastes has shown promise in maintaining material strength while minimizing CO_2_ emissions [5]. Research suggests that geopolymers, when used as a substitute for Portland cement-based products, can reduce CO_2_ emissions by approximately 9% to 64% [6], offering a more sustainable solution for the construction sector.

The sustainable development of the construction sector now heavily focuses on the use of renewable and eco-friendly materials. In this context, the utilization of natural fibers as reinforcement materials in composites, serving as an alternative to conventional fibers, is receiving increasing attention [7].

The raw material processing industry generates significant amounts of solid waste as well as environmentally harmful nitrogen and sulfur oxides. In recent years, there has been a significant increase in the use of fibers to construct highly durable structures. Natural fibers present a viable alternative to synthetic fibers, offering a distinct advantage in achieving zero-waste sustainability goals [8]. Unlike synthetic fibers, natural fibers are renewable, biodegradable, non-toxic, and possess attractive mechanical properties. However, due to their hydrophilic nature, they tend to absorb moisture, which can lead to poor matrix wettability and weak fiber–matrix interfacial bonding [9].

Geopolymer materials have emerged as a promising solution for reducing greenhouse gas emissions associated with the cement industry. Compared to traditional Portland cement, geopolymers offer a more sustainable alternative but tend to exhibit brittle fracture behavior under flexural and tensile stresses. Consequently, fiber reinforcement is required to control crack propagation, enhance toughness, and improve deformation capacity. As a result, the development of fiber-reinforced geopolymer composites (FRGCs) has attracted increasing research interest [10].

FRGCs are gaining recognition as environmentally friendly materials that can replace cement in the construction sector. Alternative inorganic binders, such as geopolymer matrices, support environmental awareness by significantly reducing CO_2_ emissions. Thus, the inorganic geopolymer matrix is considered a more eco-friendly cement alternative for FRGCs [11].

Geopolymer materials exhibit significantly lower CO_2_ emissions and environmental impact while offering comparable technical properties to Portland cement. Their superior thermal and mechanical properties make them suitable for use as clean insulation materials. Moreover, the incorporation of natural fibers such as bamboo, flax, hemp, and jute into geopolymer matrices can enhance tensile and flexural strength, reduce density, and improve thermal and acoustic insulation properties [12].

Cement-based composites offer several advantages, including high compressive strength and durability [13]. However, due to their low tensile strength, they are prone to cracking, posing structural risks [14]. To overcome this issue, cement matrices are commonly reinforced with fibers to prevent crack formation [15]. Additionally, plant-based fibers are emerging as viable alternatives to petrochemical-based materials due to their low-cost, eco-friendly production processes, and superior mechanical properties [16]. Fibers such as jute, kenaf, hemp, and bamboo are used to reinforce polymer matrix composites, finding applications in the automotive, packaging, and construction industries [17,18,19,20].

Hemp fiber (Cannabis sativa) has recently garnered significant interest for its use as reinforcement in engineering composite materials. With the growing demand for biodegradable, sustainable, and recyclable materials, the utilization of hemp fibers is becoming increasingly widespread.

Recent studies highlight the significant potential of hemp fiber-reinforced geopolymers as sustainable alternatives to conventional construction materials. For instance, lightweight geopolymer foams reinforced with hemp fibers have demonstrated enhanced thermal stability and improved resistance to brittle failure under dynamic loads. Galzerano et al.’s [21] study similarly found that adding chopped hemp fibers to a red mud/fly ash-based geopolymer matrix increased both tensile and compressive strength, while transforming the fracture behavior from brittle to pseudo-ductile. Taye et al. [22] reported that the self-treatment effect of the alkaline geopolymer environment on untreated hemp fibers has been shown to enhance fiber–matrix bonding without requiring pre-treatment, thereby improving mechanical performance and reducing processing costs.

Hemp fibers exhibit mechanical properties comparable to glass fibers; however, their variability poses a significant challenge. Composites incorporating hemp fibers reinforced with thermoplastic, thermoset, and biodegradable matrices demonstrate promising mechanical performance [23]. Various surface treatments applied to enhance fiber–matrix interfacial bonding have significantly improved the mechanical properties of these composites [24]. In the literature, numerous studies have focused on plant fiber-reinforced geopolymer composites.

Simonova et al. [25] investigated the fracture properties of mortars containing alkali-activated binders and hemp fibers. Another study examined two different types of concrete containing hemp fibers produced using (Si-Na) and (Si-Ca) reactions combined with natural aggregates. The results indicated that wet curing significantly improved the mechanical properties of the concretes, particularly with increasing cellulose content [26]. Ranjbar et al. [27] analyzed the material and geometric properties of structural fibers, as well as fiber–binder interaction mechanisms in fresh and hardened states.

Another study found that the incorporation of natural fibers such as coconut and sisal into geopolymer composites increased ductility and flexural strength by approximately 37% to 60% compared to synthetic fiber-reinforced composites. Rajendran et al. [28] and Masi et al. [29] investigated the mechanical and microstructural properties of geopolymers reinforced with PVA and basalt fibers. Additionally, the thermal and fire resistance properties of fiber-reinforced foamed geopolymers were examined. The results demonstrated that PVA fibers significantly enhanced the flexural strength and toughness of geopolymer composites, while basalt fibers improved the flexural behavior of the composites after exposure to high temperatures.

The aim of this study is to address the knowledge gaps regarding the use of hemp fibers in cement and geopolymer mortar applications in the construction sector and to bridge the gaps in the existing literature. Current research lacks sufficient insight into optimizing these fibers for structural applications. Therefore, this study aims to comprehensively investigate the practical applications of hemp fibers in construction materials.

## 2. Materials and Methods

### 2.1. Materials

The main materials used in the production of geopolymer mortars included granulated blast furnace slag (BFS) obtained from Cement Factory as the precursor material and standard sand conforming to EN 196-1 standards [30], which was used as the aggregate.

The chemical composition of the BFS used in the experimental studies is presented in Table 1. As seen, the BFS contained high levels of SiO_2_ and CaO, contributing to its excellent binding properties. It also contains various oxides, which enhance the overall performance of the geopolymer mortar. The physical properties include a density of 2.89 g/cm^3^, a Blaine specific surface area of 3940 cm^2^/g, and a moisture content of 0.5%.

Hemp fiber used as an additive element in geopolymer composites is a plant-based material consisting of approximately 70% cellulose, 15% hemicellulose, 10% lignin, and 5% pectin. The behavior and resistance of hemp fibers used in this study against different external factors are given in Table 2.

**Figure 1:**
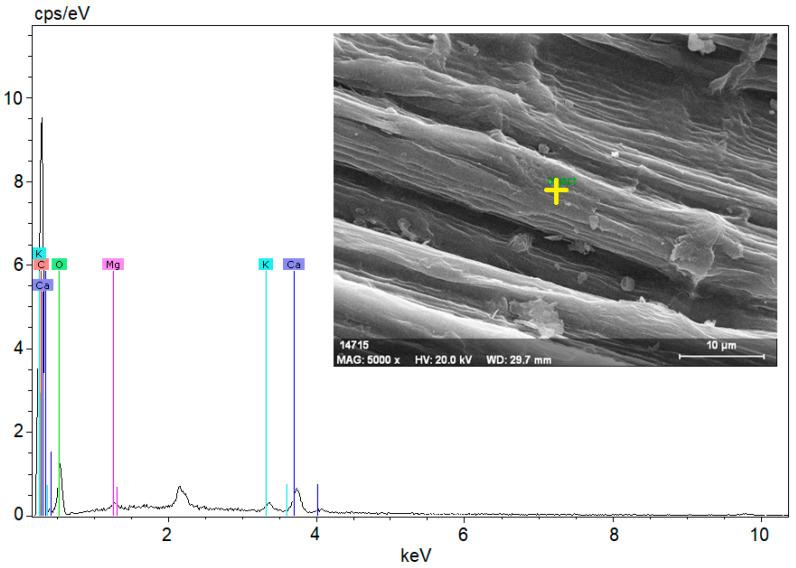
SEM image of a hemp fiber showing its surface morphology and fibrous structure, along with the EDX spectrum displaying its elemental composition.

When the literature on the thermal properties of hemp fibers is examined, it is seen that the approximate thermal conductivity is 0.039 W/mK and the specific heat capacity is 1700 J/kgK [31]. It is also stated that structural degradation begins at temperatures of 230–240 °C [32]. EDX analysis was performed together with SEM imaging to examine the microstructure and element distribution of hemp fibers used in this study. Figure 1 shows the structure of hemp fiber captured using scanning electron microscopy (SEM) and the results of elemental analysis obtained through energy-dispersive X-ray spectroscopy (EDX).

Figure 1 shows the structure of a hemp fiber captured using SEM and the results of EDX for elemental analysis. The SEM image reveals the surface structure and morphology of hemp fibers, which appear organized in smooth and elongated structures, highlighting their natural cellular structure and fibrous properties. The magnification indicated on the image is 5000×, with an accelerating voltage of 20.0 kV.

The EDX spectrum displays peaks corresponding to various elements. Carbon (C), as the primary component of the organic structure, is abundant in hemp fibers. Oxygen (O) is present in cellulose and other organic compounds within the fibers. Magnesium (Mg) is commonly found in plant cells. Chlorine (Cl) is a naturally occurring element in plants and soil. Potassium (K) is essential for plant growth and development, while calcium (Ca) contributes to cell wall structure and overall plant stability.

These elements reflect the structural and chemical properties of hemp fibers, providing important insights into their potential applications. For instance, high levels of carbon and oxygen indicate a dominant cellulose structure, supporting the biodegradable and environmentally friendly characteristics of hemp fibers. The presence of these elements in the spectrum demonstrates the chemical composition of hemp fibers and their derivation from natural, biological sources. Such analyses are critical for evaluating the potential industrial applications and suitability of hemp fibers for bio-composite materials.

### 2.2. Preparation of Geopolymer Mortars

In the alkali activator solution used for the mortar preparation, the molecular silicate modulus (Ms)—defined as the mass ratio of SiO_2_ to Na_2_O—was fixed at 0.5. The water-to-binder and sand-to-binder ratios were also kept constant at 0.5 and 3.0, respectively. The total water content in the mixture was calculated by accounting for the intrinsic water present in the alkali activator components.

The mixing process followed a standardized protocol to ensure homogeneity and optimal geopolymerization. Initially, ground granulated blast furnace slag (BFS) and standard sand were dry-blended at a low speed (136 rpm) for 30 s. During this stage, the sodium silicate solution and mixing water—containing dissolved sodium hydroxide (NaOH)—were gradually added. A rest period of 90 s followed, allowing initial precursor dissolution. Mixing then resumed at a higher speed (281 rpm) for 60 s, followed by a final 15 s rest.

Sodium hydroxide pellets with 99% purity served as the primary alkali activator. Their high alkalinity promoted the dissolution of silicon (Si) and aluminum (Al) species from the aluminosilicate precursors, effectively initiating the geopolymerization reaction and leading to the formation of the binder matrix. The sodium silicate solution used in the mix contained 26.5% SiO_2_, 8.3% Na_2_O, and 62% distilled water.

To improve the workability and performance of the geopolymer mortar, a polycarboxylate-based superplasticizer was incorporated at a dosage of 1–2% by the weight of the binder, as recommended by the manufacturer. This admixture enhanced the fresh-state flow characteristics, reduced the required water content, and contributed to achieving a denser and more durable final structure.

Following casting, the fresh mortar specimens were sealed to prevent moisture loss and cured under controlled conditions at 60 °C for 24 h in an oven, in accordance with widely accepted practices in geopolymer research [33,34]. This thermal curing step promotes rapid geopolymerization and the early development of mechanical strength. After oven curing, the samples were demolded and stored at ambient laboratory conditions (~23 ± 2 °C, 50–60% relative humidity) until the testing age. These curing conditions were selected to balance reactivity, minimize drying shrinkage, and ensure the durability of the geopolymer matrix.

In this study, the effects of adding hemp fibers in varying proportions to mortar mixes were investigated. Hemp fibers were added at volumetric percentages of 0%, 5%, 10%, and 20%, replacing other components of the mortar. This approach was employed to assess the potential benefits of hemp fibers on the mechanical properties and sustainability of mortar. Table 3 presents the mixture ratios used in the produced geopolymer composite mortars.

### 2.3. Test Procedures

#### 2.3.1. Flow Diameter

**Figure 2:**
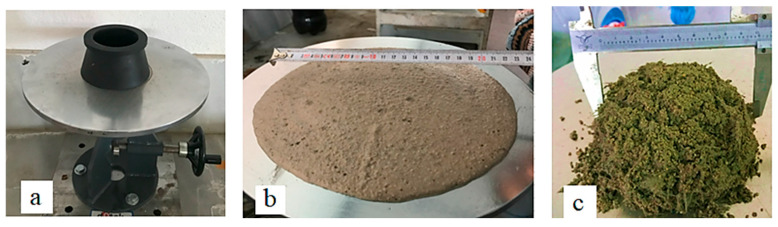
Images related to spread diameter tests: (**a**) spread diameter test device, (**b**) control sample, (**c**) geopolymer mortar with 20% HF substitution.

The ASTM C1437 [35] standard specifies a test method used to measure the flowability or spread diameter of self-compacting geopolymer mortar. This test is typically conducted by placing a quantity of the material into a flow cone and then lifting the cone to allow the material to freely spread on a flat surface. The spread diameter of the material is measured, and this measurement is considered a criterion for evaluating the fluidity of the material. Figure 2 illustrates images related to the measurement of spread diameter tests.

#### 2.3.2. Compression and Flexural Test

**Figure 3:**
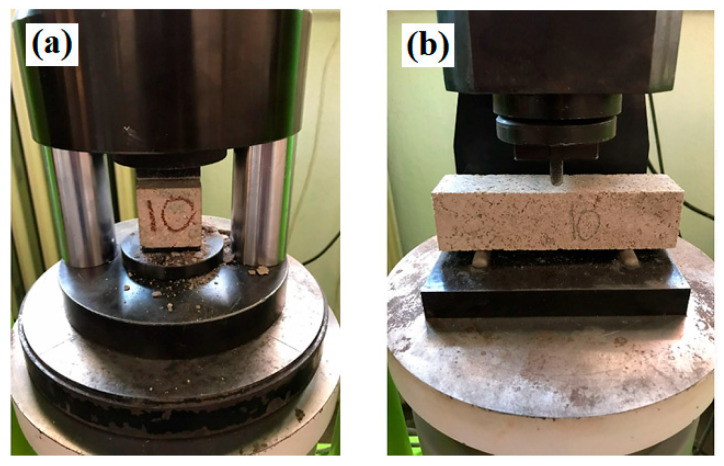
Images related to compression (**a**) and flexural tests (**b**).

The samples used in the experiments were prepared according to ASTM C348 [36] and ASTM C349 [37] standards. The samples were cast in steel molds with dimensions of 40 mm × 40 mm × 160 mm. After casting, the samples were processed with a mold release agent and subjected to mechanical vibration for 3 min. At the designated testing time for each formulation, the samples were subjected to flexural and compressive strength tests. For the flexural test, three samples were taken from each formulation and tested according to ASTM C349 standards. Similarly, for the compressive strength test, six samples were taken from each formulation and tested according to ASTM C348 standards. Figure 3 shows images related to compression and flexural tests.

#### 2.3.3. Water Absorption of Geopolymer Mortar Composite Mortars

Samples measuring 50 mm × 50 mm × 50 mm were prepared according to ASTM C 642 [38] standard for the experiments. These samples were used to investigate the water absorption characteristics of geopolymer mortar composites. Water absorption tests were conducted to determine the absorption properties of the samples after 7 and 28 days. These tests were performed to evaluate the water permeability and resistance of geopolymer mortar composites against water ingress.

The results obtained provide important information about the water absorption characteristics and durability of the mortars, contributing to the evaluation of their performance.

#### 2.3.4. Chloride Permeability of Geopolymer Composite Mortars

**Figure 4:**
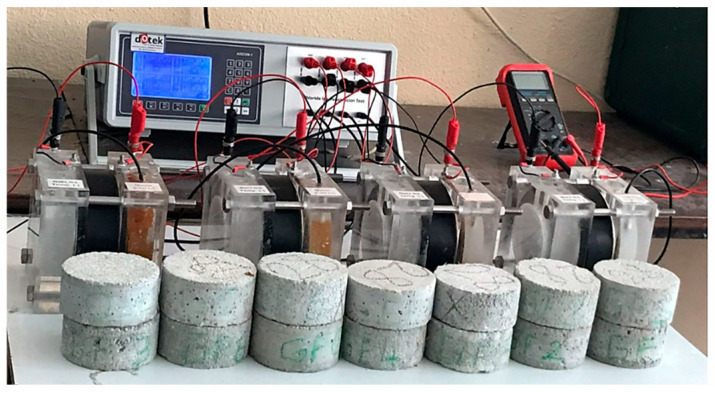
Chloride permeability test setup and samples of the mortars.

For chloride permeability, AAS mortars were prepared in Ø100 × 50 mm dimensions and subjected to chloride permeability testing. The rapid chloride ion penetration values of the samples were determined according to ASTM C 1202 [39]. A photograph taken during the chloride permeability tests is shown in Figure 4.

#### 2.3.5. Capillary Absorption Test

**Figure 5:**
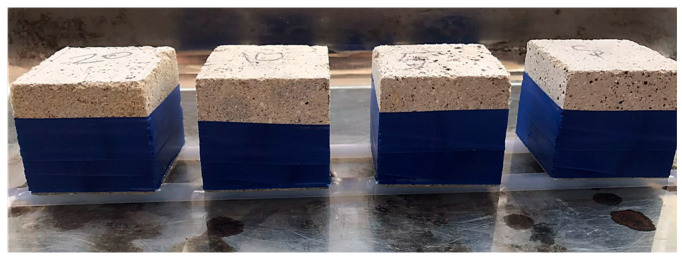
Capillary absorption test image.

The capillary absorption test is conducted to measure the movement of water in capillary pores of a material. It is commonly used to determine the water absorption and permeability properties of materials such as mortar, stone, and wood. These tests provide information about the material’s water resistance, crack resistance, and overall performance. The capillary absorption test is typically conducted according to the ASTM C1585 [40] standard. An image related to the capillary absorption test is shown in Figure 5.

#### 2.3.6. Thermal Insulation Test

**Figure 6:**
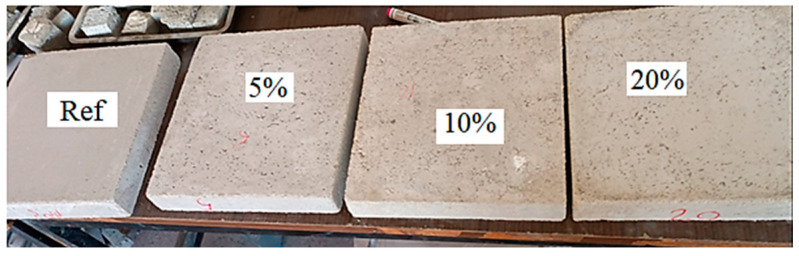
Thermal insulation test samples.

Thermal conductivity tests were conducted to determine the thermal insulation properties of the produced geopolymer mortars. The thermal conductivity values (Lambda λ) of hemp fiber-reinforced geopolymer samples were measured using a device in the temperature range of 15 to 25 °C. The conductivity measurements were performed on geopolymer samples prepared with dimensions of 300 × 300 × 50 mm. Figure 6 shows the experimental samples used for thermal conductivity measurements.

#### 2.3.7. Microstructure Analysis

SEM imaging and EDX analyses of the geopolymer mortar samples were conducted using a scanning electron microscope (Carl Zeiss, Oberkochen, Germany) at 30 kV. The samples were coated with gold to prepare the surfaces for imaging and analysis.

Phase analysis was performed using the X-ray diffraction (XRD) method to examine the phase structures of geopolymer samples. The analyses were performed using an XRD diffractometer, with Cu Kα radiation (λ = 1.5406 Å), to study the crystal structures and phase transformations of the samples. Data were collected in the 2θ range of 5–70°, with a step size of 0.02° and a scanning speed of 0.5 s/step. Prior to analysis, the hardened geopolymer mortar samples were crushed and ground to a fine powder and sieved through a 75 µm mesh to ensure uniform granulometry and minimize preferred orientation.

The XRD analysis was conducted on samples after 28 days of curing to allow sufficient time for geopolymerization and phase development. To halt further reactions and stabilize the hydration state, the powdered samples were treated with isopropanol immersion for 24 h, followed by vacuum drying at 40 °C for 48 h before analysis.

## 3. Findings and Discussion

### 3.1. Flowability and Workability

**Figure 7:**
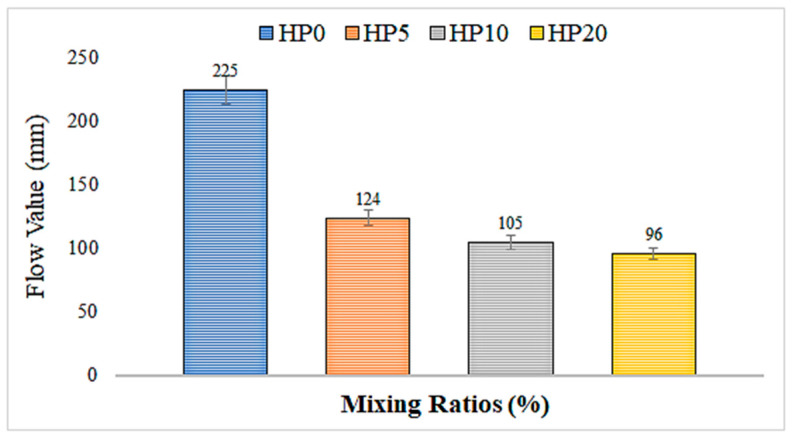
Spread value of hemp fiber-reinforced geopolymer mortar.

A graph based on the values obtained from the spread tests is presented in Figure 7. According to Figure 7, it is observed that increasing the hemp fiber ratio significantly reduces the spread value of the mortar. With the addition of 5% hemp fiber, a decrease of 22.5% occurs, with 10% addition, a decrease of 34.4% occurs, and with 20% addition, a decrease of 40% occurs.

This reduction indicates that the addition of hemp fiber to the mix reduces the fluidity and, consequently, the workability of the mortar. In a study conducted by B. Çomak et al. [41], the flow diameter results of fresh mortars were compared with the reference sample, and it was observed that the length of the hemp fiber had no effect. However, as the fiber length increased, it became more difficult to place the mortars into molds. It was explained that as the percentage of fibers increased, the flow of fresh mortars was adversely affected, and the reason for this was the increased water absorption amount with the increase in the proportion of hemp fibers.

### 3.2. Compressive Strength

**Figure 8:**
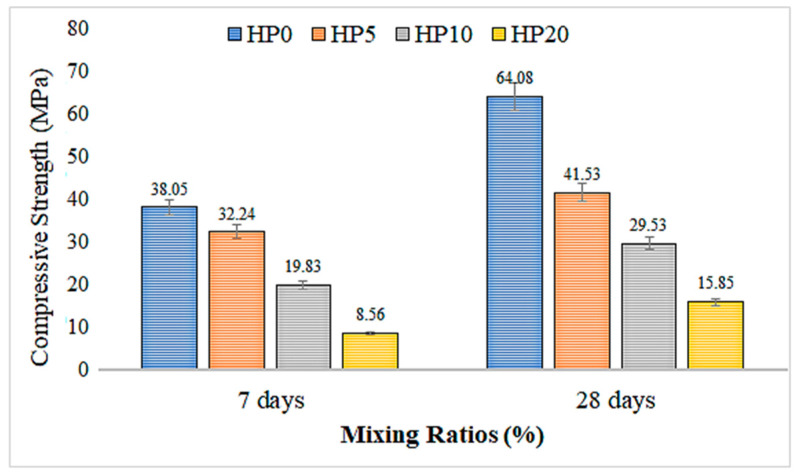
The 7-day and 28-day compressive strength results of geopolymer mortar with hemp fiber substitution.

Figure 8 presents the 7-day and 28-day compressive strength results and their standard deviations for geopolymer mortar mixtures with different hemp fiber ratios. The HP0 mixture, which does not contain hemp fiber, exhibited the highest compressive strength values at both 7 days (38.05 MPa) and 28 days (64.08 MPa), indicating superior structural performance compared to the fiber-reinforced counterparts.

As the hemp fiber content increased to 5%, 10%, and 20%, the compressive strength of the geopolymer mortar significantly decreased. Specifically, at 7 days, the compressive strengths dropped to 32.24 MPa (HP5), 19.83 MPa (HP10), and 8.56 MPa (HP20), corresponding to reductions of approximately 15.3%, 47.9%, and 77.5%, respectively, compared to the control. At 28 days, the compressive strength values were 41.53 MPa (HP5), 29.53 MPa (HP10), and 15.85 MPa (HP20), showing reductions of 35.2%, 53.9%, and 75.3%, respectively.

These results indicate that the incorporation of hemp fibers leads to a substantial decrease in compressive strength. While fiber reinforcement can be beneficial in improving tensile and flexural properties, the addition of hemp fibers appears to hinder the compressive performance of the geopolymer matrix. This reduction may be due to weak fiber–matrix bonding, inadequate dispersion, or increased porosity caused by fiber agglomeration.

Furthermore, Xie et al. [42] highlighted that the addition of fibers could improve the flexibility and tensile properties of geopolymer composites. However, at higher fiber volumes, the bond between the fiber and matrix becomes less efficient, resulting in the observed reduction in compressive strength. This is due to the increased porosity and poor dispersion of fibers within the matrix

Additionally, studies by Nath and Sarker [43] found that curing conditions play a significant role in the development of geopolymer strength. They noted that while early compressive strength may be lower in fiber-reinforced geopolymer mortars, adjusting curing conditions—such as using thermal curing—could potentially mitigate the negative impact on strength. The slow but consistent increase in strength observed between the 7-day and 28-day results for the fiber-reinforced mortars in this study supports this notion, suggesting that with improved curing techniques, further strength improvements may be possible.

A study by Hadi et al. [44] also supports the notion that while fibers can improve certain properties like flexural and tensile strengths, they tend to decrease the compressive strength in some cases. Hadi et al. observed similar trends where fiber reinforcement led to an overall reduction in compressive strength when used in certain concentrations within geopolymer matrices, which aligns with the results found in this study.

The compressive strength of this mix, used as the control group, shows the highest values in both the 7-day and 28-day measurements. This indicates that the mix without hemp fiber has a higher structural strength compared to the others. With the increase in the hemp fiber ratio, the compressive strength of the concrete has significantly decreased [45,46]. The mixes with 5%, 10%, and 20% fiber have lower strength values compared to the control group. The similar rate of strength development observed across all mixes between the 7-day and 28-day measurements suggests that the geopolymer composites continue to develop strength progressively over time, particularly under dry curing conditions [37,38].

The fibers, especially at higher concentrations, have been observed to introduce microstructural changes, leading to weaker fiber–matrix bonding and reduced strength [47]. This trend is similar to that in our results, where increased hemp fiber content (5%, 10%, and 20%) led to a progressive reduction in compressive strength.

The addition of hemp fiber has significantly reduced the strength of the mortar. This significant decrease may suggest that hemp fibers form weak bonds within the mortar matrix or are not properly homogenized [48]. As the hemp fiber ratio increases, there is a noticeable decrease in the compressive strength of the mortar. In a study conducted by Theresa Sullins et al. [49], the flexural and tensile properties of composites with 30% fiber by weight were evaluated and compared with the results of composites with 15% fiber by weight. They reported that composites with 30% fiber by weight had higher tensile and flexural properties than pure HP and, as expected, composites with 15% fiber by weight.

In conclusion, the significant reduction in compressive strength observed in this study with the addition of hemp fibers is not an isolated occurrence but aligns with the findings of other researchers. The negative impact on compressive strength, particularly at higher fiber contents, highlights the importance of optimizing fiber–matrix interactions and curing conditions in order to balance the improvements in tensile and flexural strength with the need for compressive strength. Future investigations should aim to explore these factors further, particularly the enhancement of fiber dispersion and the use of advanced curing techniques, to improve the overall performance of hemp fiber-reinforced geopolymer mortars.

### 3.3. Flexural Strength

**Figure 9:**
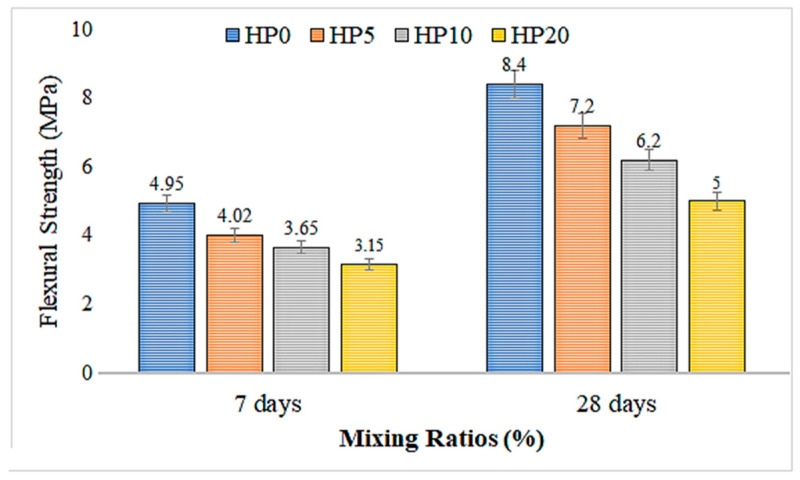
The 7-day and 28-day flexural strength results of geopolymer mortar with hemp fiber substitution.

Figure 9 presents the 7-day and 28-day flexural strength results and their standard deviations for mortar mixtures with different hemp fiber ratios. Figure 9 illustrates the flexural strength values of geopolymer mortar mixtures with varying hemp fiber content at 7 and 28 days. Similarly to the compressive strength trend, control mixture HP0, which contains no hemp fiber, exhibited the highest flexural strength values at both curing ages—approximately 4.95 MPa at 7 days and 8.4 MPa at 28 days. With the inclusion of hemp fibers, a reduction in flexural strength was observed across all mixes at 7 days. The strength values decreased to approximately 4.05 MPa (HP5), 3.65 MPa (HP10), and 3.15 MPa (HP20), indicating a gradual decline with increasing fiber content.

However, at 28 days, the effect of hemp fiber on flexural strength showed a relatively milder decline compared to the compressive strength trend. The HP5, HP10, and HP20 mixtures achieved 7.2 MPa, 6.2 MPa, and 5.0 MPa, respectively. These results suggest that while the initial integration of fibers may weaken the early-age flexural properties, the long-term flexural behavior improves over time under dry curing conditions. As the hemp fiber ratio increases, both the 7-day and 28-day flexural strengths decrease consistently. This decrease demonstrates that hemp fibers negatively affect the strength properties of mortar mixtures. In the study by D. Sedan et al. [50], the results of the flexural tests on composites showed that the fiber content varied between 0% and 20%, reaching maximum flexural strength at 16% fiber content. While the increase in fiber content up to 16% enhanced the material strength, above this ratio, the strength decreased due to inhomogeneous mixtures and weak adhesion. The composite with the optimal fiber content provided 40% more flexural strength compared to cement alone, though a 35% reduction in stiffness was observed. In summary, they noted that the fiber network increased flexural strength.

In another study, Na Lu and Shubhashini Oza [51] observed a strong correlation between the increase in fiber load and the increase in tensile and flexural strength. For hemp and new high-density polyethylene (vHDPE) composites, it was found that fiber volume fractions of 30% were optimized for both high tensile and flexural strength. However, the tensile strength of hemp and recycled high-density polyethylene (rHDPE) composites reached a maximum value of 60.2 MPa at 40% fiber load, showing a 34% improvement compared to hemp–vHDPE composites.

### 3.4. Water Absorption Test Results

**Figure 10:**
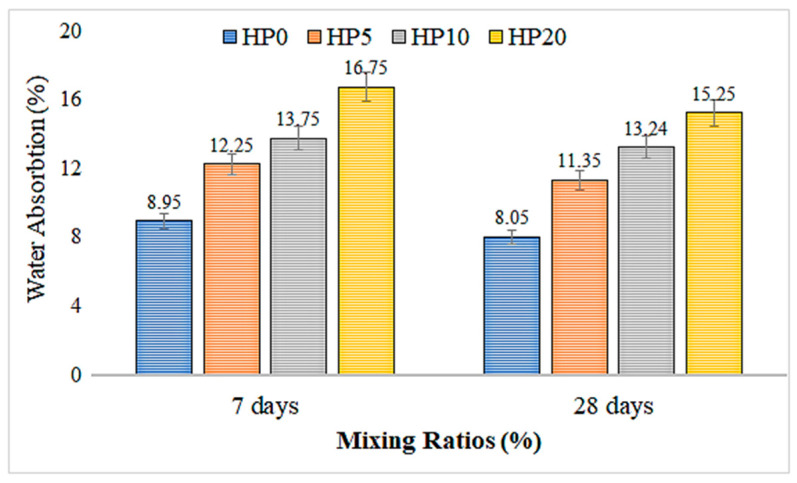
Water absorption test results of hemp fiber-reinforced geopolymer mortar.

Figure 10 depicts a graph constructed based on the data obtained from water absorption tests of geopolimer composite mortars. Figure 10 depicts the water absorption rates of mortar mixtures with different proportions of added hemp fiber over 7-day and 28-day periods. The addition of hemp fibers significantly increased the water absorption rates of the mortar mixtures. The mixture containing 5% hemp fiber (HP5) exhibited water absorption rates of 12.25% at 7 days and 11.35% at 28 days, showing a marked increase compared to the control group (HP0). When the fiber ratio was increased to 10% (HP10), the water absorption rates were measured at 13.75% at 7 days and 13.24% at 28 days, recording higher values compared to the control group. In the mixture with the highest fiber ratio (HP20), the water absorption rate was observed to be 16.75% at 7 days and 15.25% at 28 days, indicating a significant increase in water absorption capacity with an increasing fiber ratio.

While all mixtures containing hemp fibers exhibited higher water absorption rates compared to the control group, these rates decreased over time. This decrease is associated with the maturation process of the mortar. Initially, hemp fibers have a high water absorption capacity [52], but this capacity decreases over time. The ability of hemp fibers to increase water absorption rates results in increased porosity of the mortar, allowing more water to be absorbed. These findings highlight the need to carefully consider the use of hemp fiber-reinforced mortar in structures requiring water insulation. However, the thermal insulation properties and eco-friendly characteristics offered by hemp fibers support their preference in sustainable construction projects.

### 3.5. Geopolymer Composite Mortars Chloride Permeability

**Figure 11:**
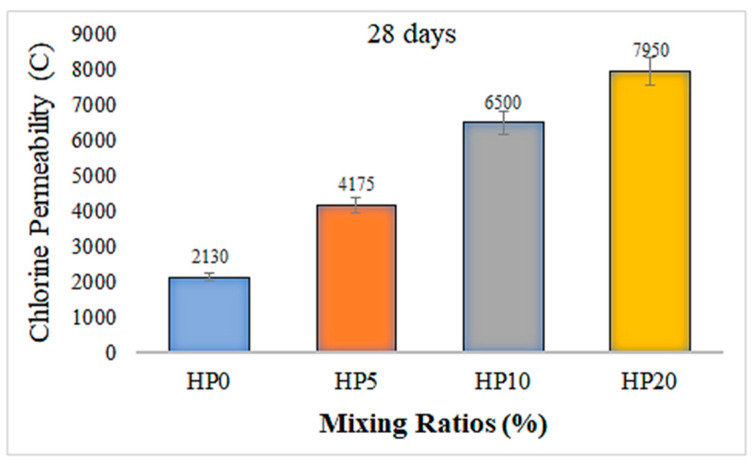
Chloride permeability of geopolymer composite mortars.

Figure 11 presents the graph constructed based on the data obtained from chloride permeability tests of geopolymer composite mortars. Figure 11 illustrates the chloride permeability results of geopolymer composite mortars containing various ratios of hemp fiber after 28 days of curing. The results reveal a significant increase in chloride ion permeability with the incorporation of hemp fibers.

The control mixture without any fiber addition (HP0) exhibited the lowest charge passed value of 2130 Coulombs, indicating a dense microstructure with limited pore connectivity, which effectively restricts chloride ion penetration. However, as the hemp fiber content increased to 5%, 10%, and 20%, the total charge passed increased progressively to 4175 Coulombs, 6500 Coulombs, and 7950 Coulombs, respectively. This trend suggests that the inclusion of hemp fibers leads to a more porous matrix structure, likely due to poor fiber dispersion, microcracking, or insufficient bonding between the fiber and the geopolymer matrix. The observed increase in chloride permeability with higher fiber content indicates a potential compromise in the long-term durability of the geopolymer mortars, particularly in aggressive environments where chloride ingress is a critical concern, such as marine or de-icing conditions. Despite the potential benefits of natural fiber inclusion in terms of sustainability and environmental performance, the results demonstrate that excessive fiber content may deteriorate the impermeability of the composite. Therefore, optimization of fiber content and improvements in fiber–matrix interfacial bonding are necessary to maintain a balance between mechanical performance and durability in hemp fiber-reinforced geopolymer systems. It has also been observed in the study that the chloride permeability results are consistent with the 28-day compressive strength findings.

### 3.6. Capillary Absorption Test Results

**Figure 12:**
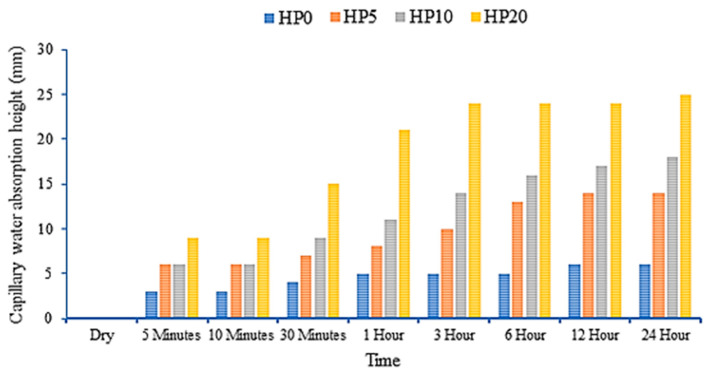
Capillary absorption test results of hemp fiber-reinforced geopolymer mortar.

Figure 12 shows the graph constructed based on the capillary absorption test results of hemp fiber-reinforced geopolymer composites. Analyses indicate that the water absorption amounts of the samples increase over time, demonstrating that the water absorption capacities of the samples reach saturation over time. Particularly within the 24 h period, a significant increase in water absorption values is observed for the majority of the samples. As the fiber content in the samples increases, there is an increase in water absorption amounts.

The sample containing 20% fiber notably absorbs more water compared to the other samples, indicating that the fiber enhances water absorption properties and increases the material’s water retention capacity. At each hourly interval, an increase in water absorption rates is observed as the fiber content increases. For instance, the sample with 20% fiber exhibits a higher water absorption percentage at different time intervals such as 5 min, 10 min, and 1 h compared to the other samples, highlighting that the fiber enhances both the rate and amount of water absorption.

The sample with 5% fiber shows an increase in water absorption over time. However, its water absorption percentage is lower compared to the samples with 10% and 20% fiber. Hourly comparisons generally indicate that the sample with 5% fiber tends to have a lower water absorption percentage compared to the other samples, suggesting that the sample with 5% fiber has a lower water absorption capacity than the others and that fiber concentration directly affects water absorption ability.

The sample with 10% fiber exhibits a higher water absorption capacity than the sample with 5% fiber but lower than the sample with 20% fiber. An increase in water absorption amounts over time is observed, although it has a lower water absorption percentage compared to the sample with 20% fiber. Hourly comparisons indicate that the sample with 10% fiber generally has a higher water absorption percentage compared to the sample with 5% fiber.

These analyses elucidate the water absorption behaviors of different fiber concentrations. It is concluded that the sample with 5% fiber has the lowest water absorption capacity, the sample with 10% fiber increases this capacity, and the sample with 20% fiber generally exhibits the highest water absorption capacity. These findings underscore the significant role of fiber content in influencing water absorption properties.

### 3.7. Thermal Conductivity

**Figure 13:**
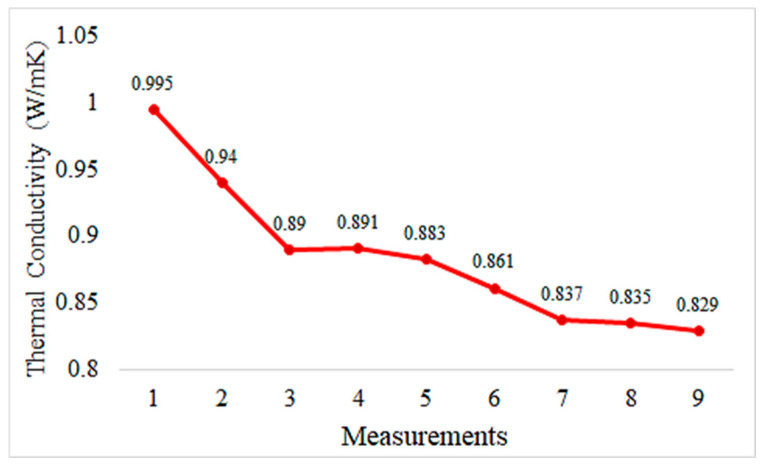
Thermal conductivity values of the control sample.

**Figure 14:**
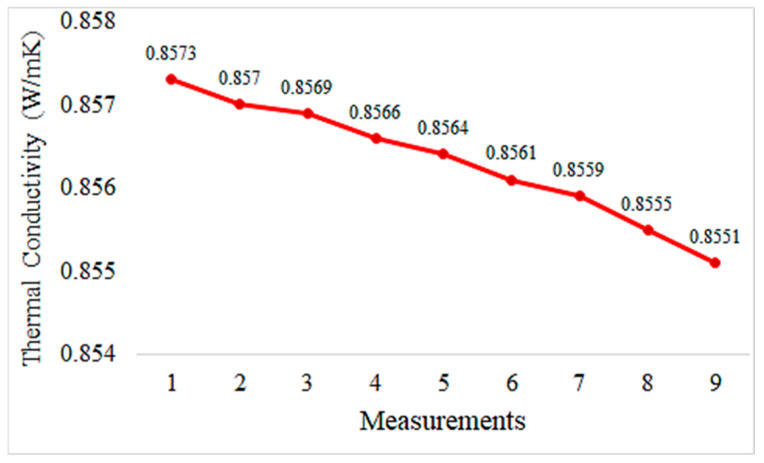
Thermal conductivity measurement values of geopolymers reinforced with 5% hemp fiber.

**Figure 15:**
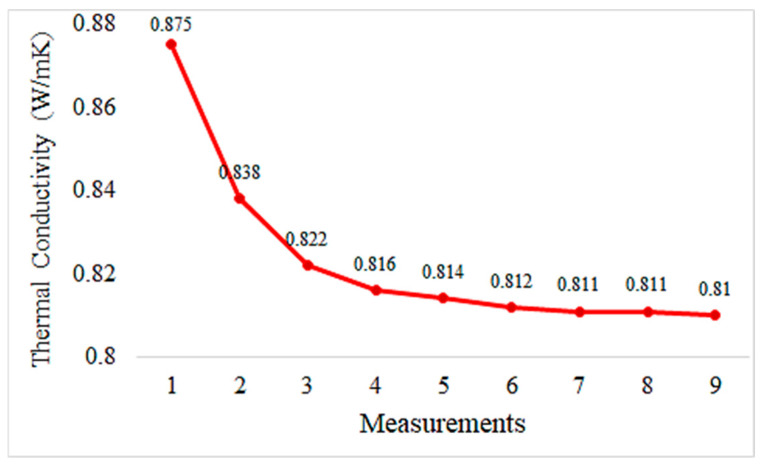
Thermal conductivity measurement values of geopolymers reinforced with 10% hemp fiber.

**Figure 16:**
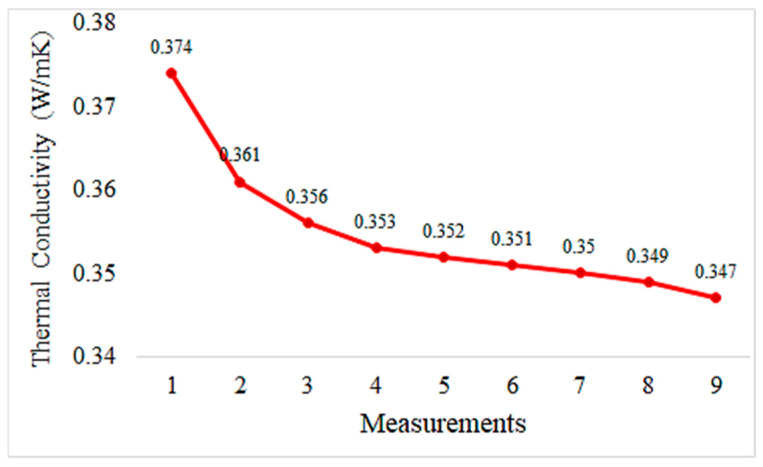
Thermal conductivity measurement values of geopolymers reinforced with 20% hemp fiber.

**Figure 17:**
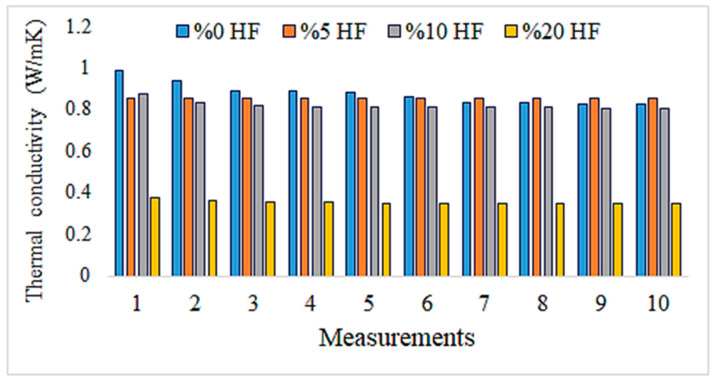
Thermal conductivities of geopolymer composites reinforced with 0%, 5%, 10%, and 20% hemp fiber.

Graphs depicting thermal conductivity measurements of geopolymer mortar plates with different proportions of HF additives and the reference without additives are provided in Figure 13, Figure 14, Figure 15, Figure 16 and Figure 17. The graphs are presented both individually for each sample and collectively for detailed comparison. Ten measurements were taken at different times for each sample.

The thermal conductivity values represent geopolymers reinforced with 5% hemp fiber. These values appear to remain relatively stable over time. This indicates that the thermal conductivity of the mortar is consistent and homogeneous. Generally, a stable and low thermal conductivity suggests that the mortar is a good thermal insulating material. Compared to the control sample, the addition of hemp fiber reinforcement enhances the thermal insulation properties of the mortar. The incorporation of hemp fiber contributes to making the mortar a better thermal insulating material.

Upon reviewing the data in Figure 15, we observe that it is lower than hemp fiber-reinforced mortar at 5%. This shows that 10% hemp fiber reinforcement increases the thermal insulation properties of the mortar and decreases them less than 5% hemp fiber reinforcement.

The values indicate that compared to the mortar reinforced with 10% hemp fiber, this is lower. This indicates that 20% hemp fiber reinforcement enhances the mortar’s thermal insulation properties and reduces them less than 10% hemp fiber reinforcement.

Having lower thermal conductivity values for mortar reinforced with 20% hemp fiber indicates that this reinforcement enhances the mortar’s thermal insulation properties. This can improve the energy efficiency of building materials and may be a preferred option for sustainable construction. The graph below shows the thermal conductivity values for all geopolymers together.

**Figure 18:**
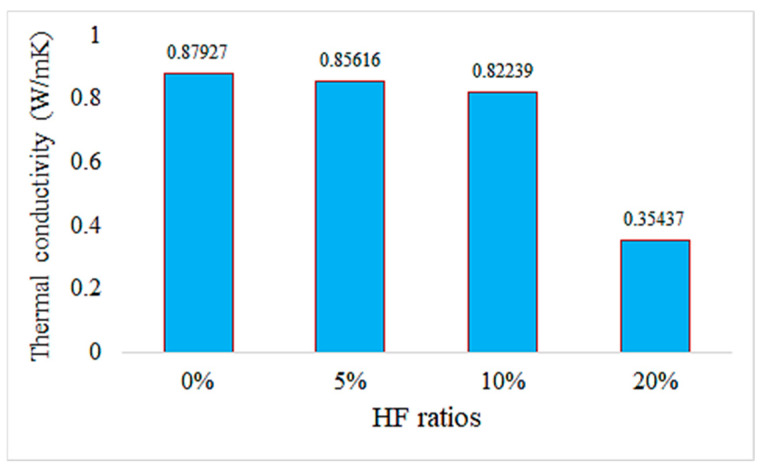
Thermal conductivity values of hemp fiber composites.

The thermal conductivity values of hemp fiber-reinforced mortar specimens generally decrease with increasing HF content. This indicates that hemp fiber reinforcement can enhance the thermal insulation properties of mortar. The thermal conductivity values of the specimen with 20% HF content are significantly lower than for the other specimens, suggesting that higher HF content may improve the thermal insulation performance of mortar. The control specimen represents the thermal conductivity properties of mortar without hemp fiber reinforcement. When compared to the other specimens, hemp fiber-reinforced mortars generally exhibit lower thermal conductivity values. These findings demonstrate that hemp fiber-reinforced mortar has the potential to enhance thermal insulation performance and can be used as environmentally friendly building materials. To further evaluate the thermal conductivity behavior of composite pastes, arithmetic averages of ten thermal measurements were taken for each specimen and plotted accordingly in Figure 18.

In Figure 18, it is observed that as the HF ratio increases, the thermal conductivity values generally decrease. This indicates that hemp fiber reinforcement can enhance the thermal insulation properties of mortar. Particularly noteworthy is that the sample with a 20% HF ratio exhibits significantly lower thermal conductivity compared to the other samples, suggesting that higher HF ratios can improve the thermal insulation performance of mortar.

The control sample (0% HF) represents mortar without hemp fiber reinforcement. Compared to the other samples, hemp fiber-reinforced mortars generally demonstrate lower thermal conductivity values.

These results indicate that hemp fiber-reinforced mortar has the potential to enhance thermal insulation performance and can be considered environmentally friendly building materials. Sair et al. also obtained similar results in their study and reported that the use of hemp fiber in building insulation materials is promising [53].

The addition of hemp fiber has been shown to significantly enhance the thermal insulation properties of mortar. Notably, samples containing 20% hemp fiber (HF) exhibited much lower thermal conductivity values compared to the control sample without any fiber reinforcement. This indicates that increasing the HF ratio leads to better insulation performance. Supporting this, Parcesepe et al. [7] found that lime mortar reinforced with hemp fibers demonstrated substantially reduced thermal conductivity, outperforming conventional concrete in insulation performance. Similarly, McGinn et al. [54] showed that applying hemp–lime renders to solid brick walls halved the thermal transmittance (U-value), bringing performance on par with commercial insulating materials like autoclaved aerated concrete. Furthermore, Audouin et al. [55] demonstrated that replacing synthetic fibers with natural hemp fibers in structural mortars not only improved environmental impact but also resulted in better thermal insulation properties. These findings strongly support the conclusion that hemp fiber-reinforced mortars provide superior insulation compared to traditional materials and should be considered sustainable and eco-friendly alternatives in the construction industry.

### 3.8. Microstructure Evaluation and Phase Analysis

**Figure 19:**
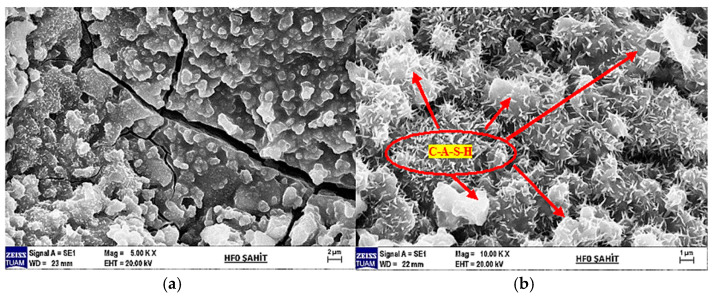
(**a**) SEM image of the witness sample at 5000× magnification and (**b**) at 10,000× magnification.

**Figure 20:**
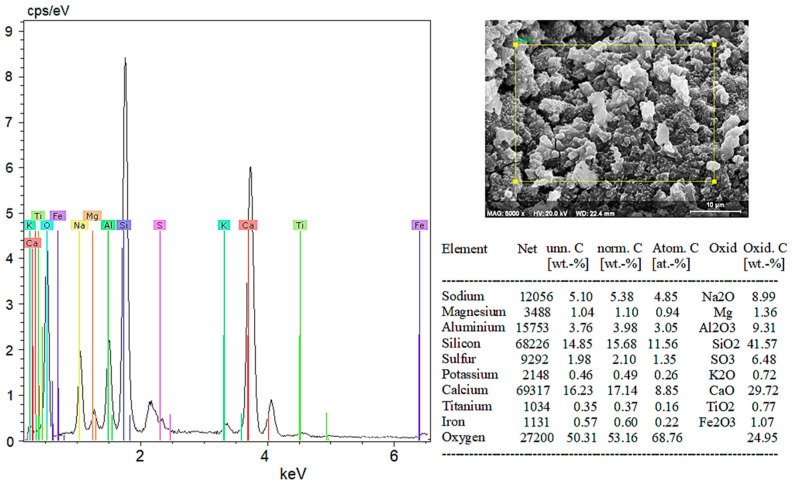
EDX results of the witness sample.

**Figure 21:**
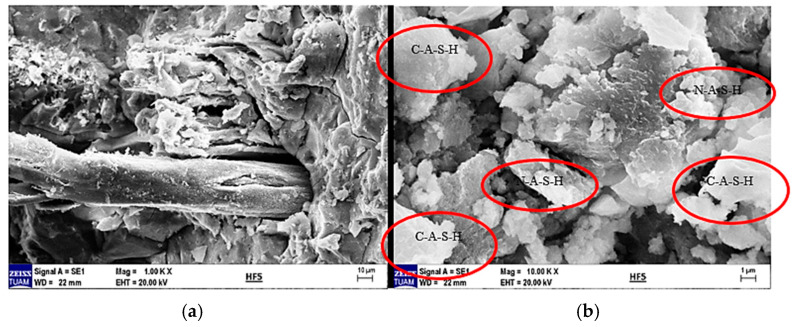
(**a**) SEM image of 5% hemp fiber-reinforced geopolymer mortar at 250× magnification and (**b**) at 1000× magnification.

**Figure 22:**
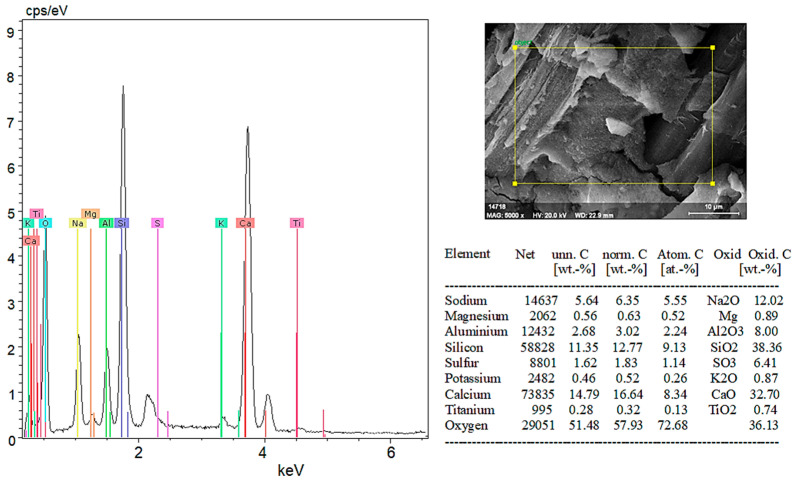
EDX analysis results of 5% hemp fiber-reinforced geopolymer mortar.

**Figure 23:**
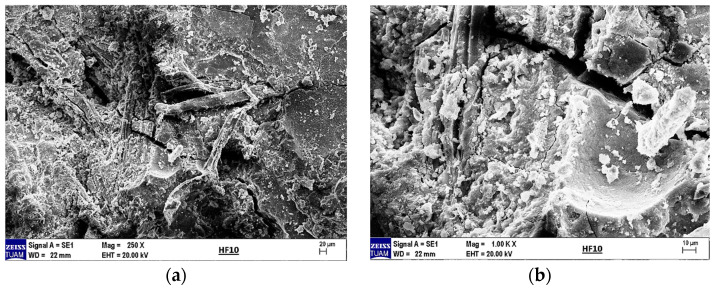
(**a**) SEM image of 10% hemp fiber-reinforced geopolymer mortar at 250× magnification and (**b**) at 1000× magnification.

**Figure 24:**
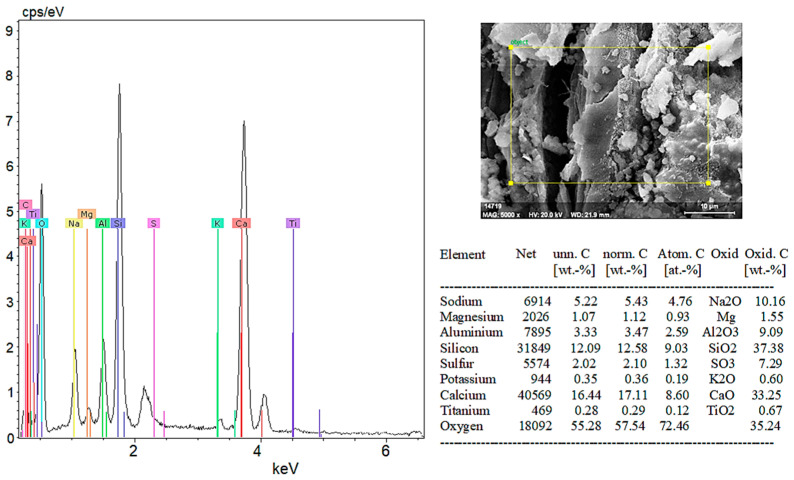
EDX analysis results of 10% hemp fiber-reinforced geopolymer mortar.

**Figure 25:**
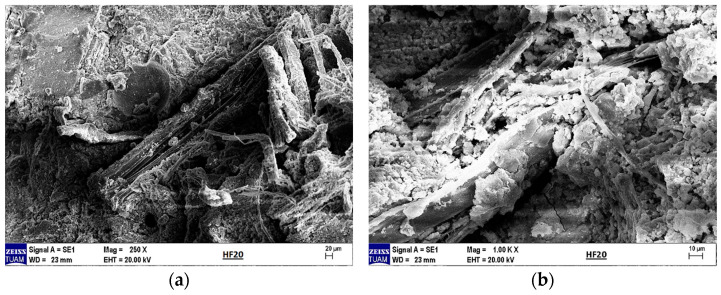
(**a**) SEM image of 20% hemp fiber-reinforced geopolymer mortar at 250× magnification and (**b**) at 1000× magnification.

**Figure 26:**
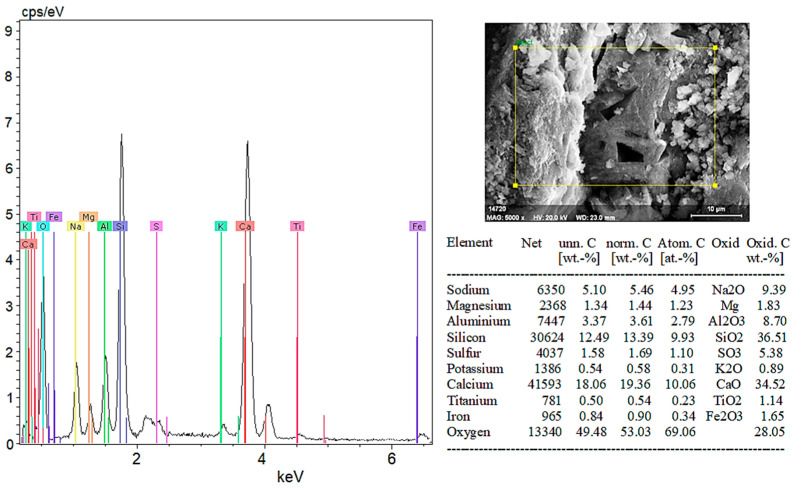
EDX analysis results of 20% hemp fiber-reinforced geopolymer mortar.

SEM images were taken at different magnifications to examine the internal structures of hemp fiber-reinforced geopolymer composites. Additionally, EDX elemental analyses were conducted for each sample. The following section evaluates these images and analyses, presented in Figure 19, Figure 20, Figure 21, Figure 22, Figure 23, Figure 24, Figure 25 and Figure 26.

In Figure 19a at 5000× magnification, with EHT (Acceleration Voltage) of 20.00 kV, the SEM image reveals prominent cracks and a rough surface texture. These features highlight the material’s microstructural properties and surface deteriorations.

In Figure 19b at 10,000× magnification, with EHT (Acceleration Voltage) of 20.00 kV, the SEM image provides a more detailed view of the surface. This image allows for clearer observation of the particles and microstructure on the surface, which are crucial for understanding the composition and microstructure of the material.

According to the EDX analysis results in Figure 20:

- Sodium (Na) and Potassium (K): Sodium and potassium are alkali metals commonly found in plants and natural minerals, indicating the material may have organic origins.
- Magnesium (Mg) and Calcium (Ca): These elements are frequently found in plant cell walls and natural minerals. A high proportion of calcium suggests calcium compounds (CaO) are significant components of the material’s structure.
- Aluminum (Al) and Silicon (Si): Aluminum and silicon are typically found in clay minerals and natural soil components. A high percentage of SiO_2_ indicates the material is rich in silica.
- Sulfur (S): The presence of sulfur indicates the presence of sulfates (SO_3_) in the material.
- Titanium (Ti) and Iron (Fe): These elements are usually present in low amounts in mineral and soil components.

In conclusion, SEM and EDX analyses provide a detailed analysis of both organic and inorganic components of the hemp fiber-reinforced geopolymer composite (witness) sample. These analyses are critical for understanding the material’s microstructural and chemical properties. The significantly high content of calcium and silicon in the hemp fiber-reinforced sample supports its potential use as a durable structural material.

In Figure 21a, the SEM image of 5% hemp fiber-reinforced geopolymer mortar at 1000× magnification, and (b) at 10,000× magnification, provides a detailed examination of the surface of geopolymers. These images clearly reveal particles and microstructures on the surface, highlighting the heterogeneous nature of the geopolymer matrix. The distribution of hemp fibers within the matrix and the differences in microstructures formed around the fibers are prominently observable. Particles observed around the fibers indicate the formation of chemical bonds at the fiber–matrix interface, contributing to the overall mechanical strength of the material.

The C-A-S-H phase typically exhibits an amorphous or semi-crystalline structure. Therefore, in the SEM image, it appears as a more homogeneous and continuous structure rather than distinct crystalline formations. The C-A-S-H phase is usually dense and compact, appearing darker and smoother. It is frequently observed in regions where bonds between hemp fibers and the geopolymer matrix are concentrated, indicating a denser structure in these areas.

N-A-S-H forms during the geopolymerization reaction through the use of alkali activators (e.g., sodium silicate). This phase enhances the chemical durability and mechanical properties of geopolymers.

C-H is known as a hydration product in Portland cement but can also be found in geopolymer systems. Excessive C-H may not be desirable in geopolymers as it is susceptible to carbonation and other chemical reactions over time, potentially compromising the material’s chemical durability. It forms as a result of the hydration of calcium-rich compounds and is typically found within the matrix’s microstructure.

The presence of fibers can promote a more homogeneous distribution of phases such as C-S-H and C-A-S-H within the matrix. These phases formed around the fibers can enhance the bond strength at the fiber–matrix interface, thereby increasing mechanical strength.

The SEM image of 5% hemp fiber-reinforced geopolymer mortar can clearly depict the formation and distribution of these structures mentioned above. Structures such as C-S-H, C-A-S-H, and N-A-S-H within the geopolymer matrix significantly improve the material’s durability and mechanical properties. These structures are often more densely clustered around hemp fibers, which enhances the bond strength at the fiber–matrix interface.

When examining the EDX analysis results in Figure 22, it is evident that the 5% hemp fiber-reinforced geopolymer mortar forms a robust structure through the combination of various inorganic components such as sodium and potassium as alkali elements, as well as magnesium, calcium, aluminum, silicon, sulfur, titanium, and iron.

Sodium (Na) and potassium (K), present in the form of Na_2_O and K_2_O, act as alkali activators that initiate and sustain the geopolymerization reaction. These oxides enhance the chemical durability of the matrix by promoting the dissolution and rearrangement of aluminosilicate species. Magnesium (Mg) and calcium (Ca), commonly introduced via MgO and CaO, play key roles in improving the mechanical properties of the material. In particular, calcium contributes to the formation of additional calcium silicate hydrate (C–S–H) phases, which enhance compressive strength and density. Aluminum (Al) and silicon (Si), present primarily as Al_2_O_3_ and SiO_2_, are the fundamental building blocks of geopolymer binders. High silicon content facilitates the development of a well-connected aluminosilicate network, crucial for matrix formation and long-term stability. Sulfur (S), typically found as SO_3_, can contribute to sulfate resistance by reacting with calcium to form stable ettringite-like phases. Trace elements such as titanium (Ti) and iron (Fe) influence the color, density, and other physical properties of the composite.

In conclusion, the 5% hemp fiber-reinforced geopolymer sample exhibits a strong structure formed by the combination of various inorganic components. The addition of hemp fibers improved the material’s mechanical and chemical properties. Such analyses are critical for evaluating the suitability of geopolymers for environmental and structural applications. The addition of hemp fiber can enhance the material’s sustainability and environmental friendliness.

The SEM image of 10% hemp fiber-reinforced geopolymer mortar can clearly depict the formation and distribution of the structures mentioned above. Within the geopolymer matrix, structures such as C-S-H, C-A-S-H, and N-A-S-H significantly enhance the material’s durability and mechanical properties. These structures are more densely clustered around hemp fibers, which have strengthened the bond at the fiber–matrix interface.

According to the EDX analysis in Figure 24, the chemical composition of 10% hemp fiber-reinforced geopolymer mortar is as follows: Na_2_O (sodium oxide), 10.16%; Mg (magnesium), 1.55%; Al_2_O_3_ (aluminum oxide), 9.09%; SiO_2_ (silicon dioxide), 37.38%; SO_3_ (sulfur trioxide), 7.29%; K_2_O (potassium oxide), 0.60%; CaO (calcium oxide), 33.25%; and TiO_2_ (titanium dioxide), 0.67%. Oxygen constitutes 35.24% of the total oxide compounds.

These compositions clearly illustrate the main components of the geopolymer matrix and their distribution on the surface. In particular, at high content, SiO_2_ and CaO are fundamental components that enhance the mechanical and chemical durability of geopolymer mortar. The appropriate distribution of these elements positively influences the overall performance and durability of the material.

When examining Figure 25, the SEM images of 20% hemp fiber-reinforced geopolymer mortar reveal a dense distribution of fibers within the matrix. The fibers are uniformly dispersed within the geopolymer matrix and have formed a strong bond at the fiber–matrix interface. This bonding enhances the material’s mechanical strength. Dense C-A-S-H (Calcium Aluminosilicate Hydrate) phases are observed around the fibers. The C-A-S-H phase typically exhibits an amorphous or semi-crystalline structure and appears more homogeneous and continuous in the SEM images. This phase densification around the fibers enhances the chemical durability and dimensional stability of the matrix.

The substitution of hemp fibers increases the formation of microporous structures, which enhances the material’s permeability and chemical resistance. The porous structure observed around the fibers improves the performance characteristics of the mortar. Particles formed around the fibers indicate the formation of chemical bonds, contributing to the overall mechanical strength of the material. Areas with intense bonding at the fiber–matrix interface are clearly observable in these images.

In 20% hemp fiber-reinforced geopolymer mortar, a heterogeneous matrix structure is observed, which increases the material’s flexibility and limits crack propagation. Compared to 5% and 10% fiber-reinforced mortars, the higher concentration of fibers in 20% hemp fiber substitution results in a denser and more homogeneous distribution of fibers within the matrix. This leads to more distinct and intense phase changes in the microstructure compared to lower fiber percentages used previously. The higher fiber content increases the porosity of the matrix, resulting in a more pronounced formation of microporous structures compared to 5% and 10% substitution rates.

The 20% hemp fiber substitution leads to a denser formation of C-A-S-H and N-A-S-H phases within the matrix. These phases, particularly concentrated around the fibers, enhance the chemical durability and mechanical properties of the mortar. The higher fiber content ensures a more intense chemical bonding at the fiber–matrix interface than observed in 5% and 10% fiber-reinforced mortars, thereby significantly increasing the material’s mechanical strength.

SEM images of 20% hemp fiber-reinforced geopolymer mortar clearly highlight significant changes and improvements in the material’s microstructure. The high fiber content promotes the densification of C-A-S-H and N-A-S-H phases, enhancing the material’s durability and establishing strong bonds at the fiber–matrix interface, thereby positively impacting the overall performance of the geopolymer mortar. It is crucial to determine appropriate fiber ratios and ensure the homogeneous distribution of these phases to optimize the chemical and physical performance of geopolymer mortar.

According to Figure 26, the chemical composition of 20% hemp fiber-reinforced geopolymer mortar, based on EDX analysis, is as follows: Na_2_O (sodium oxide), 9.39%; Mg (magnesium), 1.83%; Al_2_O_3_ (aluminum oxide), 8.70%; SiO_2_ (silicon dioxide), 36.51%; SO_3_ (sulfur trioxide), 5.38%; K_2_O (potassium oxide), 0.89%; CaO (calcium oxide), 34.52%; TiO_2_ (titanium dioxide), 1.14%; and Fe_2_O_3_ (iron oxide), 1.65%. Oxygen is present at 28.05% in the total oxide compounds. This composition clearly indicates the main components of the geopolymer matrix and their distribution on the surface. In particular, at high contents, SiO_2_ and CaO are essential components that enhance the mechanical and chemical durability of geopolymer mortar. The proper distribution of these elements positively impacts the overall performance and durability of the material.

**Figure 27:**
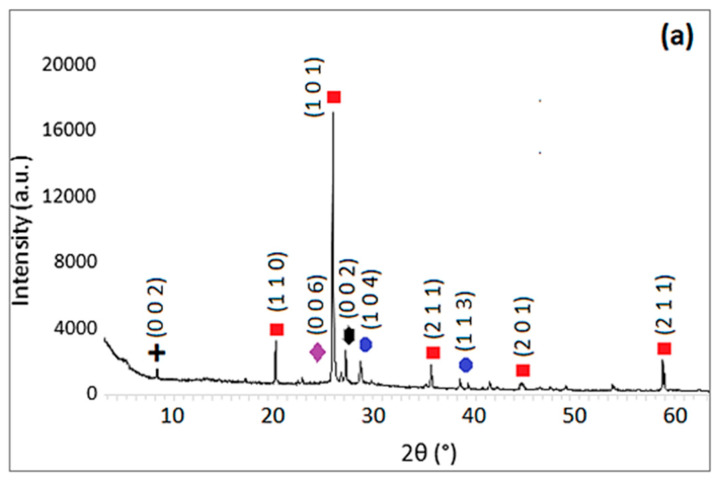

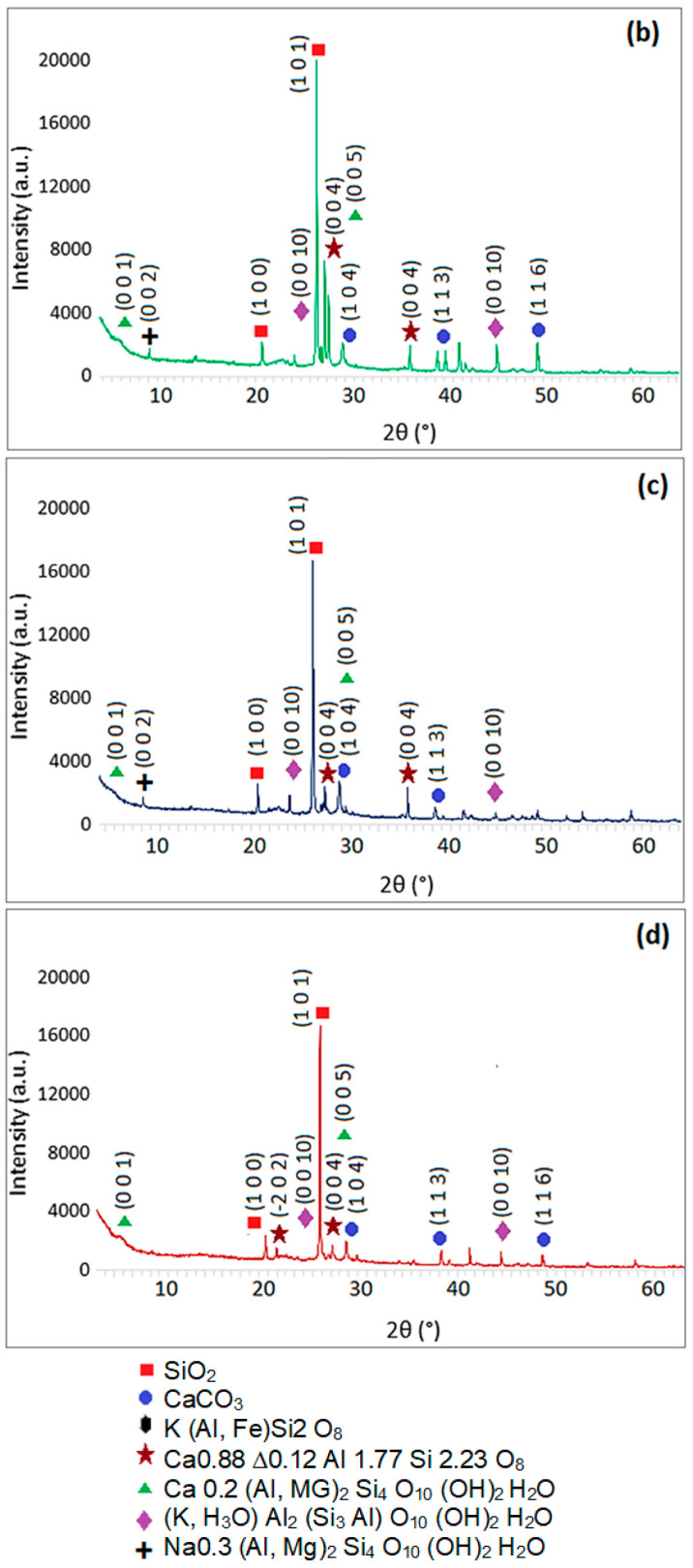
XRD analysis results of 0% (**a**), 5% (**b**), 10% (**c**), and 20% (**d**) hemp fiber-reinforced geopolymer mortar.

Graphs created according to the data obtained from the XRD (X-ray diffraction) analysis of hemp fiber-reinforced composite materials are given in Figure 27.

When examining the XRD graphs of the 0% (reference), 5%, 10%, and 20% HF-reinforced samples in Figure 27, it is clear that quartz constitutes the dominant phase in all the samples. The quartz phase shows the most intense peak in the (101) plane at 26.63° 2θ, belonging to the Hexagonal P3121 (152) crystal structure. This phase also exhibits other crystal planes corresponding to Miller indices (h k l) at 20.83°, 36.52°, 39.45°, 50.16°, and 59.98° 2θ. This phase structure was consistent across all composite samples. The primary reason for the dominance of the quartz (SiO_2_) phase observed in the XRD analysis is the use of standard sand conforming to EN 196-1, which contains 97.22% SiO_2_ by chemical composition, a significant portion of which is present in the crystalline quartz form.

Following quartz, the next significant phase is calcite. The most intense peak of the calcite phase is observed in the Rhombohedral R-3c (167) crystal structure at 29.40° 2θ in the (104) plane. Other peaks associated with this phase are found at 39.40°, 43.14°, and 48.51° 2θ, corresponding to Miller indices (h k l) of (113), (202), and (116), respectively. The presence of calcite is primarily attributed to the blast furnace slag (BFS) used in the composites, which contains 35% CaO. Therefore, the XRD analyses reveal quartz and calcite as the most prominent mineralogical phases in all the composite samples.

The third most notable phase identified in the geopolymer composite samples is anorthite, a type of calcium aluminosilicate. This phase’s presence is influenced by the primary components of the composites, BFS and silica sand. The most intense peak for the anorthite phase appears at 28.01° 2θ in the (004) plane of the Triclinic C (0) crystal structure. Additional peaks corresponding to this phase are observed at 22.02°, 23.60°, and 27.76° 2θ, with Miller indices (h k l) of (−202), (112), and (040).

Further examination of the XRD patterns reveals the presence of various other minor phases in the geopolymer composite structures. While the peaks for these phases are less intense, they appear across all composite structures. These secondary mineral phases include potassium aluminum silicate hydrates, sodium aluminum silicates, and calcium magnesium aluminum silicate hydrates. These are indicated by low-intensity diffraction peaks and are typical in geopolymer systems due to the diverse raw material composition. The elements and compounds detected in the EDX analysis of the geopolymer composite materials further support the findings from the XRD phase analysis.

According to the XRD analyses, the predominant mineral phase in all samples was identified as quartz, with the most prominent peak observed at 26.63° 2θ in the (101) plane. However, important crystalline phases such as calcite and anorthite were also detected, attributed to the high content of silica sand and BFS in the composite structure. Additionally, some secondary phases were observed, showing low-intensity peaks in the XRD analyses.

SEM and EDX analyses further supported these phase findings, especially in highlighting the effects of fiber addition on the microstructure. In the control sample (0% HF), SEM images showed distinct cracks and a rough surface texture, while EDX analysis confirmed high amounts of Si and Ca, supporting the presence of quartz and calcite phases identified in the XRD analyses. In the 5% HF-added sample, SEM images showed an even distribution of fibers within the matrix, with C-A-S-H phases concentrated at the fiber-matrix interface, exhibiting darker and more homogeneous appearances due to their amorphous nature. EDX analysis revealed high levels of Ca, Si, Al, and Na, corroborating the chemical structure and showing that the fibers facilitated the formation of these binding phases, in line with the work of Temuujin et al. [56] and Fernandez-Jimenez et al. [57]. For the 10% HF-added sample, the EDX analysis revealed a composition of 37.38% SiO_2_ and 33.25% CaO, confirming the chemical presence of the dominant mineral phases identified in the XRD analysis. These ratios indicate high mechanical strength and a denser microstructure, as noted by Ma et al. [58]. The 20% HF-added sample demonstrated a more intense and homogeneous fiber distribution, with C-A-S-H and N-A-S-H phases becoming more prominent in these areas. This enhanced the chemical bonding at the fiber–matrix interface, thereby improving the material’s mechanical strength. Additionally, the increased micro-porosity of these phases contributed to improved chemical durability, as observed by Poinot et al. [59]. Overall, the combined evaluation of SEM, EDX, and XRD analyses clearly demonstrates the positive effects of hemp fiber addition on the mineral phase formation, microstructure, and chemical durability of geopolymer mortars. The fiber incorporation, especially through the concentration of C-A-S-H and N-A-S-H phases, enhances both mechanical and chemical performance. These findings align with the work of Deb et al. [60] and Ma et al. [61], supporting the potential of geopolymers as sustainable building materials.

## 4. Conclusions

This study extensively investigated the effects of hemp fiber addition on various properties of geopolymer mortar. The findings indicate the significant impacts of hemp fibers on the workability, compressive strength, water absorption capacity, and thermal conductivity properties of the geopolymer mortar:

1. Slump Value of Geopolymer Mortar:

Increasing hemp fiber content significantly reduces the slump value of geopolymer mortar. There is a reduction of approximately 22.5% with 5% hemp fiber addition, 34.375% with 10%, and 40% with 20% addition. This indicates that fibers reduce the fluidity and workability of the geopolymer mortar.

2. Compressive Strength:

The control mix without hemp fibers shows the highest compressive strength values at both 7 and 28 days. As the hemp fiber content increases, the compressive strength of geopolymer mortar decreases significantly. Mixes with 5%, 10%, and 20% fibers exhibit lower strength values compared to the control group. In particular, the mix with 20% fibers has only about 24% of the compressive strength of the control group.

3. Flexural Strength:

The flexural strength of the control group also shows the highest values. Increasing hemp fiber content results in a decrease in the flexural strength of the geopolymer mortar. With increasing fiber content, both 7-day and 28-day flexural strength values consistently decrease.

4. Water Absorption Capacity:

The water absorption of samples increases over time. A significant increase in the water absorption rate is observed with increasing fiber content. The sample with 20% fiber absorbs more water compared to other samples, indicating that fibers enhance the water absorption properties and water holding capacity of the material. The sample with 5% fiber shows an increase in water absorption over time, but it is lower compared to samples with 10% and 20% fiber.

5. Thermal Conductivity:

Hemp fiber reinforcement improves the thermal insulation properties of geopolymer mortar. Thermal conductivity values remain close to constant with 5% hemp fiber addition and decrease further with 10% and 20% hemp fiber addition. This suggests that hemp fibers enhance the thermal insulation performance of geopolymer mortar, contributing to energy efficiency.

6. Microstructural and Chemical Analyses:

SEM and EDX analyses provide detailed insights into the distribution and composition of organic and inorganic phases within the geopolymer mortar matrix. The results indicate that hemp fibers are not uniformly dispersed, leading to potential weak zones and negatively impacting mechanical strength. However, the formation of reaction products such as C-S-H, C-A-S-H, and N-A-S-H around the fibers suggests a partial reinforcement effect, which may enhance durability and mechanical properties under specific conditions. These findings offer a more comprehensive understanding of the microstructural role of fibers in geopolymer composites and highlight areas for further optimization.

In XRD analysis, the most effective phases in the composite structures were SiO_2_, Ca-CO_3_, and CaAl_2_Si_2_O_8_, respectively. The most important reason for this was found to be the silica sand and blast furnace slag used in the formation of geopolymer composite structures. In addition, it was determined that low levels of Potassium Aluminum Silicate Hydroxide Hydrate, Potassium Aluminum Silicate, Sodium Aluminum Magnesium Silicate, Sodium Aluminum Silicate, Sodium Aluminum Magnesium Silicate Hydroxide Hydrate, and Calcium Magnesium Aluminum Silicate Hydroxide Hydrate phases were present in all composite samples.

In conclusion, hemp fiber reinforcement reduces the workability and mechanical strength of geopolymer mortar but enhances water absorption capacity and thermal insulation properties. These findings suggest that hemp fiber-reinforced geopolymer mortar can be used in specific applications, particularly for thermal insulation and sustainable building materials. However, careful optimization of fiber content is crucial for applications where mechanical strength is critical.

## Figures and Tables

**Table 1:** Chemical and physical properties of BFS.

Oxides (wt.%)	SiO_2_	Al_2_O_3_	Fe_2_O_3_	CaO	MgO	Na_2_O	K_2_O	TiO_2_	SO_3_	Mn_2_O_3_	CI
BFS	34.82	17.51	0.68	34.13	5.41	0.42	1.71	1.10	0.69	3.52	0.01
Physical Properties	Blaine fineness (cm^2^/g)				Loss on Ignition (%)			Density (g/cm^3^)			
BFS	3940				1.84			2.89			

**Table 2:** Resistance of hemp fiber to external factors.

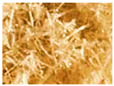	**Weather**	**Acid**	**Alkali**	**Solvent**	**Aging**	**Light**
	Bad	Moderate	Good	Good	Bad	Bad

**Table 3:** Mix ratios of geopolymer composite mortar.

Samples	Fiber (%)	BFS (g)	NaOH (g)	Na_2_SiO_3_ (g)	Sand (g)	Water (g)
**HF0**	0	810	57.6 *	228.6 *	2430 *	262.8 *
**HF 5**	5	769.5				
**HF 10**	10	729				
**HF 20**	20	648				

* This value is the same in all samples.

## Data Availability

The original contributions presented in this study are included in the article. Further inquiries can be directed to the corresponding author.
